# Advances in Developing Medical Students’ ‘Web-side Manner’

**DOI:** 10.1177/23821205211064692

**Published:** 2025-02-27

**Authors:** Ophelia Millar, Gledisa Musollari

**Affiliations:** Imperial College London School of Medicine, London, UK

**Keywords:** medical education, medical curriculum, Covid-19

Dear Editor,

We read the article “Medical Students and Patients Benefit from Virtual Non-Medical Interactions Due to COVID-19”^1^ by *T. Coe* with great interest. The pilot study introduced the concept of non-medical, semi-structured virtual student-patient interactions without time constraints or specific agendas.^1^ In this letter, we would like to add to the development of the student curriculum to include more virtual-based consultation teaching by offering the perspective of medical students who have experienced medical teaching both before and during the COVID-19 pandemic. Alongside outlining the positive impact T. Coe's work had on the development of the medical curriculum, we will discuss additional ideas which we believe could further benefit the development of the medical curriculum. We agree that experiential learning is crucial in the development of students’ communication skills during consultations, particularly when these consultations occur through a novel medium such as virtually or over the phone. Additionally, the NHS have stated that the increased use of virtual consultations seen during the COVID-19 pandemic is to likely continue in future, particularly in primary care, due to its ability to maintain patient experience ratings while bettering workload and time management.^2^ For this reason, the development of healthcare professionals’ ‘web-side manner’ is essential for continued safe and effective patient care.

The virtual interactions explored in *T. Coe*'s work led to the ‘expansion of clinical communication skills’, formally assessed through a clinical encounter focus group debrief,^1^ alongside increased knowledge of patient conditions and their attitudes towards said conditions.^1^ Following on from the study's success, these virtual interactions have since been incorporated into their medical school's curriculum.^1^ The students involved in this pilot study were provided with an interview guide to maintain the flow of the consultation if required, but no formal teaching on virtual communication skills were given.^1^ The semi-structured approach to the consultations allowed students freedom to guide the conversations and develop their confidence in talking to patients outside of an exam setting.^1^

Patients involved in the study were also given the opportunity to provide feedback on their experience of the student consults. Some patients highlighted the importance of appropriate communication skills, outlining previous experiences of doctors *‘assuming that* [patients] *understand things, and it's going right over their head*’.^1^ Assumptions like these are caused by poor communication skills, and thus the addition of a web-side barrier may further impact patient care.

However, in *T. Coe*'s study, no specific teaching was delivered on how to transfer in-person communication skills to virtual patient interactions. The addition of a virtual communication skills workshop prior to these interactions would have allowed for improved efficiency during the consultations, maximising the benefits of the experience for both students and patients alike. Equipping medical students with the skills to provide excellent healthcare via virtual consultations during medical school would ensure they have a holistic, adaptable approach to achieve comprehensive healthcare, both in-person and virtually, from the beginning of their careers.

As medical students, we have received high quality teaching in the importance of non-verbal communication for in-person consultation and thus we are confident in our face-to-face communication skills. Since returning to clinical studies during the COVID-19 pandemic, we feel ill-equipped to effectively communicate with patients in telephone and video consultations. Our heavy reliance on non-verbal communication during patient interactions thus becomes challenging when speaking to patients through a virtual medium. Additional teaching addressing the differences between in-person and virtual consultations, virtual non-verbal communication, and use of virtual consultation-specific software is imperative for the development of our skillset in the current digital era.

An example of virtual communication-specific teaching can be seen in a separate study conducted in the United Kingdom by *Gunner et al*^3^ This study aimed to *‘increase the knowledge and skills relating to video consultation in final year medical students’* by in-person small group teaching sessions with a remotely located patient volunteer.^3^ Students in this study had objectives clearly outlined to them prior to a witnessing an example of best practice video consultations, alongside discussion about how these differ to in-person consultations.^3^ This allowed for increased understanding and internalisation of adaptations required in digital consultations, and was then consolidated with both a simulation and reflection session.^3^ Their 7 learning outcomes were designed to enhance the virtual communication skills of medical students, with student confidence evaluation via a self-assessment questionnaire.^3^ This was completed both pre and post teaching, which found significant improvement across all 7 learning outcomes (P < 0.001).^3^

Although the aforementioned studies were conducted in different countries, and investigated different aims, we believe an amalgamation of the two could provide an exemplary format for web-side manner teaching in medical schools around the world. Academic institutions would benefit from providing students with structured teaching on achieving best practice in virtual consultations, with sufficient opportunities to consolidate these skills. We believe this is best done in an unexamined, low-pressure setting, allowing for true reflection of the experience which would aid safe and effective participate in the novel digital demands of the healthcare system. This also abides by the ‘Implementation Science’ method of learning,^4^ by instigating intrinsic considerations when acting to achieve best practice through understanding of the learning objectives prior to the experience, and through measuring the outcomes of the act by reflection post-simulation.^4^ Developing a virtual medicine curriculum in this way would produce future doctors who are competent in providing excellent healthcare in both face-to-face and virtual consultations.
